# Supplementary material for: Distribution of bacterial communities along the spatial and environmental gradients from Bohai Sea to northern Yellow Sea
Source: PeerJ. 2018 Jan 29;6:e4272. doi: 10.7717/peerj.4272 (PMC5793709; doi:10.7717/peerj.4272)
Supplement: Supplemental Information 1 [file peerj-06-4272-s001.docx]

**Figure S1** Contour maps of important environmental factors from Dataset S1. Figures A-C shows the distribution of salinity, turbidity and nitrite in surface water, while figures D-F shows the distribution of temperature, salinity and turbidity in bottom water.

**Figure S2** The most significant taxa of three clusters (Fig. 2). Discriminant taxa with a log LDA (least discriminant analysis) score > 3.9 were shown in this figure. More details are listed in Dataset S2.

**Figure S3** Bacterial alpha-diversity estimators vary among communities in surface and bottom water.

**Figure S4** Relationships between geographic distance, depth, environment and community similarity are shown in this figure. Community similarity was calculated as 1 - weighted UniFrac distance. Scatter plots were generated separately for samples from surface and bottom water. Regression lines, along with regression coefficients (R) and probability (P), were generated using general linear model (GLM).

**Figure S5** Venn plots show numbers of shared OTUs among adjoining samples from surface and bottom water.

**Table S1** Estimators of bacterial alpha-diversity are shown for each sample in the table.

**Table S2** Mantel and Partial Mantel test were performed for the Spearman's rank correlations between environmental parameters and beta-diversity using all, surface and bottom samples. Correlation coefficients (|ρ| ≥ 0.5) are highlighted with gray shadow. Significant correlations (P ≤ 0.05) are labeled with bold type. Geo-distance: geographic distance; Wat-depth: water depth; Col-depth: collection depth; Chl *a*: chlorophyll a.

**Figure S1** Contour maps of important environmental factors from Dataset S1. Figures A-C shows the distribution of salinity, turbidity and nitrite in surface water, while figures D-F shows the distribution of temperature, salinity and turbidity in bottom water.

**Figure S2** The most significant taxa of three clusters (Fig. 2). Discriminant taxa with a log LDA (least discriminant analysis) score > 3.9 were shown in this figure. More details are listed in Dataset S2.

**Figure S3** Bacterial alpha-diversity estimators vary among communities in surface and bottom water.

**Figure S4** Relationships between geographic distance, depth, environment and community similarity are shown in this figure. Community similarity was calculated as 1 - weighted UniFrac distance. Scatter plots were generated separately for samples from surface and bottom water. Regression lines, along with regression coefficients (R) and probability (P), were generated using general linear model (GLM).

**Figure S5** Venn plots show numbers of shared OTUs among adjoining samples from surface and bottom water.

**Table S1** Estimators of bacterial alpha-diversity are shown for each sample in the table.

| **Samples** | **97% Similarity** | | | | |
| --- | --- | --- | --- | --- | --- |
|  | **Observed OTUs** | **Good’s Coverage (%)** | **Phylogenetic Diversity** | **Chao1** | **Shannon-Wiener** |
| P1s | 299 | 99.5% | 31.53 | 338.00 | 6.06 |
| P1b | 289 | 99.5 | 31.32 | 331.24 | 6.12 |
| B8s | 317 | 99.5 | 34.27 | 363.88 | 5.48 |
| B8b | 350 | 0.996 | 36.55 | 378.45 | 6.52 |
| B6s | 300 | 0.994 | 33.71 | 342.98 | 5.56 |
| B6b | 349 | 0.995 | 35.78 | 380.32 | 6.03 |
| R5s | 247 | 0.994 | 27.05 | 286.08 | 5.32 |
| R5b | 350 | 0.995 | 35.80 | 371.50 | 6.10 |
| L5s | 263 | 0.994 | 28.79 | 299.43 | 5.18 |
| L5b | 371 | 0.995 | 37.57 | 401.15 | 6.49 |
| E4s | 252 | 0.995 | 27.51 | 299.52 | 5.23 |
| E4b | 329 | 0.995 | 33.88 | 372.83 | 5.98 |
| K5s | 192 | 0.996 | 22.04 | 220.96 | 4.76 |
| K5b | 252 | 0.996 | 28.58 | 283.14 | 5.88 |

**Table S2** Mantel and partial Mantel tests for the Spearman's rank correlations between environmental parameters and beta-diversity. Tests were performed separately using all samples, samples from surface water and bottom water. Correlations are shown in this table and highlighted with gray shadow when |ρ| ≥ 0.5. When P values are less than 0.05 after Benjamini-Hochberg multiple test correction, correlations are shown with bold type.

| **Samples** | **Variables** | **Mantel**  **tests** | **Partial Mantel tests** | | |
| --- | --- | --- | --- | --- | --- |
|  |  |  | **control factors** | | |
|  |  |  | **Geographic**  **distance** | **Water**  **depth** | **Collection**  **depth** |
| All samples | Temperature | **0.21** | **0.19** | 0.17 | −0.33 |
|  | pH | **0.59** | **0.59** | **0.59** | **0.53** |
|  | Turbidity | **0.30** | **0.27** | **0.31** | **0.33** |
|  | Dissolved oxygen | **0.21** | **0.24** | **0.26** | 0.18 |
|  | NO_2_-N | **0.36** | **0.36** | **0.37** | 0.28 |
|  | SiO_3_-Si | **0.19** | **0.19** | 0.19 | −0.01 |
| Samples from  surface water | Turbidity | **0.78** | **0.70** | **0.76** | **0.77** |
|  | NO_2_-N | **0.78** | **0.70** | **0.78** | **0.78** |
| Samples from  bottom water | Temperature | **0.77** | **0.67** | 0.2 | 0.22 |
|  | Salinity | **0.75** | **0.63** | 0.2 | 0.22 |
|  | PO_4_-P | **0.59** | **0.58** | 0.27 | 0.31 |
